# Bis(4,4′-bipyrid­yl)bis{2-[4,6-bis­(carboxy­methyl­sulfan­yl)-1,3,5-triazin-2-ylsulfan­yl]acetato}zinc(II)

**DOI:** 10.1107/S160053681000735X

**Published:** 2010-03-06

**Authors:** Suna Wang, Yan Yang, Dacheng Li, Jianmin Dou, Daqi Wang

**Affiliations:** a College of Chemistry and Chemical Engineering, Liaocheng University, Liaocheng 252059, People’s Republic of China

## Abstract

In the title compound, [Zn(C_9_H_8_N_3_O_6_S_3_)_2_(C_10_H_8_N_2_)_2_], the central Zn^II^ ion, situated on a center of inversion, adopts an octa­hedral geometry coordinated by four O atoms from two carboxyl­ate groups and two carboxylic groups of two symmetry-related TTTA ligands and two N atoms from two bpy mol­ecules {TTTA is 2,2′,2′′-[1,3,5-triazine-2,4,6-triyltris(sulfanedi­yl)]triacetic acid and bpy is 4,4′-bipyridine}. These mononuclear units are connected through complementary O—H⋯*X* hydrogen bonds, as well as through weak C—H⋯*X* (*X* = O and N) inter­actions, resulting in a three-dimensional supra­molecular architecture.

## Related literature

For crystal engineering of carboxyl­ates, see: Moulton & Zaworotko (2001 ▶); Rao *et al.* (2004 ▶); Ferey *et al.* (2005 ▶). For inter­actions involved in the self-assembly process, see: Braga & Grepioni (2000 ▶); Roesky & Andruh (2003 ▶); Chen *et al.* (2009 ▶). For our work on the coordination chemistry of semirigid polycarboxyl­ate ligands with functional groups introduced between the aromatic ring and carboxyl­ate groups, see: Wang *et al.* (2007 ▶); Hong *et al.* (2005 ▶); Sun *et al.* (2007 ▶). 
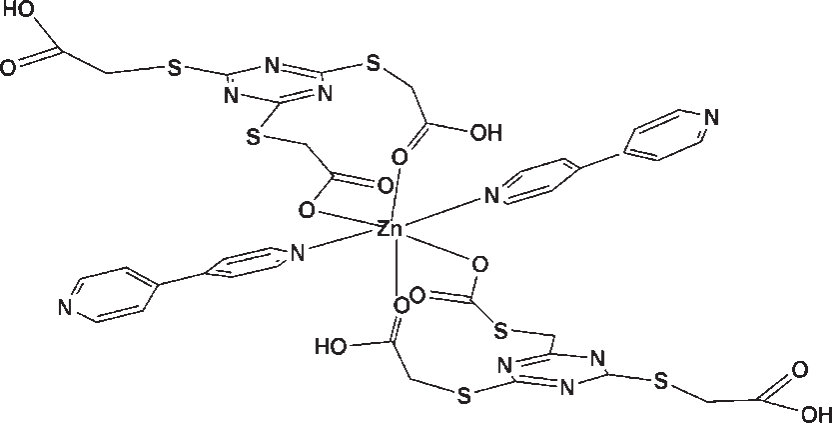


## Experimental

### 

#### Crystal data

[Zn(C_9_H_8_N_3_O_6_S_3_)_2_(C_10_H_8_N_2_)_2_]
*M* *_r_* = 1078.47
Triclinic, 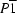

*a* = 8.6025 (7) Å
*b* = 8.7606 (7) Å
*c* = 15.3187 (12) Å
α = 99.518 (1)°
β = 105.802 (2)°
γ = 98.805 (1)°
*V* = 1071.41 (15) Å^3^
*Z* = 1
Mo *K*α radiation
μ = 0.94 mm^−1^
*T* = 293 K
0.28 × 0.24 × 0.23 mm

#### Data collection

Bruker SMART APEX CCD diffractometer
Absorption correction: multi-scan (*SADABS*; Sheldrick, 1996 ▶) *T*_min_ = 0.81, *T*_max_ = 0.84
5465 measured reflections
3745 independent reflections
3103 reflections with *I* > 2σ(*I*)
*R*_int_ = 0.067

#### Refinement

*R*[*F* ^2^ > 2σ(*F* ^2^)] = 0.041
*wR*(*F* ^2^) = 0.116
*S* = 1.05
3745 reflections
306 parameters
H-atom parameters constrained
Δρ_max_ = 0.55 e Å^−3^
Δρ_min_ = −0.63 e Å^−3^

### 

Data collection: *SMART* (Bruker, 2000 ▶); cell refinement: *SAINT* (Bruker, 2000 ▶); data reduction: *SAINT*; program(s) used to solve structure: *SHELXTL* (Sheldrick, 2008 ▶); program(s) used to refine structure: *SHELXTL*; molecular graphics: *SHELXTL*; software used to prepare material for publication: *SHELXTL*.

## Supplementary Material

Crystal structure: contains datablocks I, global. DOI: 10.1107/S160053681000735X/rn2067sup1.cif

Structure factors: contains datablocks I. DOI: 10.1107/S160053681000735X/rn2067Isup2.hkl

Additional supplementary materials:  crystallographic information; 3D view; checkCIF report

## Figures and Tables

**Table 1:** Selected bond lengths (Å)

Zn1—O3	2.1145 (19)
Zn1—N4	2.135 (2)
Zn1—O1	2.189 (2)

**Table 2:** Hydrogen-bond geometry (Å, °)

*D*—H⋯*A*	*D*—H	H⋯*A*	*D*⋯*A*	*D*—H⋯*A*
O2—H2⋯O4^i^	0.82	1.64	2.460 (3)	175
O5—H5⋯N5^ii^	0.82	1.74	2.554 (3)	174
C13—H13⋯O6^iii^	0.93	2.47	3.335 (4)	156
C19—H19⋯O6^iii^	0.93	2.34	3.245 (4)	164
C6—H6*A*⋯N1^iv^	0.97	2.58	3.533 (4)	168

Symmetry codes: (i) 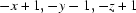; (ii) 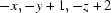; (iii) 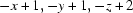; (iv) 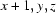.

**Fig. 1.**
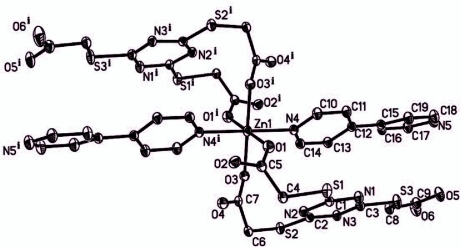
The local coordination environment for the ZnII centers in 1. Hydrogen atoms have been omitted for clarity, and thermal ellipsoids are drawn at the 30% probability level. Selected bonds information is listed in Table 1. Symmetry codes: (i) 1-x, -y, 1-z.

**Fig. 2.**
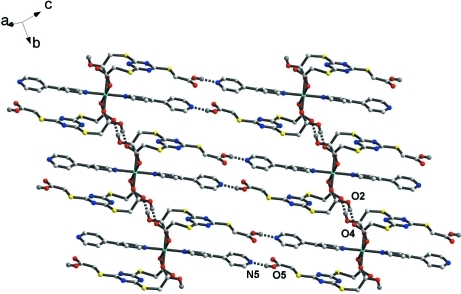
View of the two-dimensional layer, constructed by O—H···N and O—H···O hydrogen bonding between the adjacent mononuclears. Only the hydrogen atoms in hydrogen bonds are shown for clarity.

**Fig. 3.**
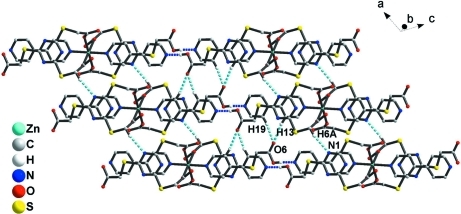
View of the C—H···O and C—H···N weak interactions between the layers. Only the hydrogen atoms in hydrogen bonds are shown for clarity.

## supplementary crystallographic information

### Comment

Recent years have witnessed rapid development of the construction of metal-organic assemblies with fascinating structures and properties in coordination chemistry and crystal engineering. (Moulton & Zaworotko, 2001; Rao *et al.*, 2004; Ferey *et al.*, 2005). Besides metal-ligand coordination bonding, various kinds of intermolecular weak interactions, such as hydrogen bonds, weak C—H···X (X = O, N, π) interactions and π···π stacking, are also vital in the self-assembly process. (Braga & Grepioni, 2000; Roesky & Andruh, 2003; Chen *et al.*, 2009) Our interest is the coordination chemistry of semirigid polycarboxylate ligands by introducing functional groups between the aromatic ring and carboxylate groups (Hong *et al.*, 2005; Wang *et al.*, 2007; Sun *et al.*, 2007).

Herein we report the title compound [Zn(TTTA)_2_(bpy)_2_] (TTTA = 2,2',2''-[1,3,5-triazine-2,4,6-triyltris(thio)]tris-acetic acid, bpy = 4,4'-bipyridine), as illustrated in Scheme 1 and Figure 1. The Zn^II^ ion, situated on a center of inversion, adopts octahedral geometry with four oxygen atoms from two carboxylate groups and two carboxylic groups of two different TTTA ligands and two nitrogen atoms from two coordinated bpy molecules. Only one carboxylate group of the TTTA ligand is deprotonated and coordinated to the metal center in a monodentate mode. The atoms in the central triazine ring are almost coplanar with a very small deviation of only 0.0085 Å from the mean plane and the dihedral angle of the carboxylate group with the triazine ring is 75.6 (2)°. The other two -COOH groups, one of which is coordinated and the other uncoordinated, form the dihedral angles of 80.0 (2) and 175.0 (4)° with the triazine ring, respectively.

As shown in Figure 2, significant O—H···N hydrogen bonding interactions are generated between hydroxyl groups (O5-H5) of the carboxylic acid and uncoordinated nitrogen atom (N5) from adjacent molecules. As a result, one-dimensional hinged chains containing M_2_L_2_(bpy)_2_ macrocyclic rings are formed along the b axis. These chains are further linked together in a parallel fashion to form a two-dimensional sheet through O—H···O hydrogen bonds between the carboxylate group (O2) and carboxyl oxygen atom (O4) from adjacent chains. Between neighboring sheets, bpy (C13 and C18) CH groups form weak C—H···O weak interactions with TTTA carboxyl oxygen atoms (O6). Simultaneously, these sheets are consolidated further through weak C—H···N interactions between CH_2_ groups (C6) and N atoms (N1) of the triazine ring (Figure 3 and Table 2). Thus, the mononuclear units are connected together through the complementary interactions of several kinds of hydrogen bonds, which ultimately extend into a three-dimensional framework.

### Experimental

A mixture of TTTA (0.025 mmol, 0.010 g), bpy (0.05 mmol, 0.008 g), Zn(NO_3_)_2_.6H_2_O (0.025 mmol, 0.013 g) with H_2_O (10 ml) was placed in a Parr Teflon-lined stainless steel vessel and heated to 80 °C for 24 h. Then the reaction system was cooled to room temperature slowly and light yellow block crystals were obtained. After filtration, the crystals were washed with water and dried in air. Elemental analysis calculated for C_38_H_32_N_10_O_12_S_6_Zn (Mr = 1078.47) : C 42.32, H 2.99, N 12.99%; found: C 42.13, H 2.92, N 13.08%.

### Refinement

All H atoms were placed geometrically and treated as riding on their parent atoms with C—H 0.93(pyridine,benzene), C—H 0.97 (methylene) Å [*U*_iso_(H) = 1.2*U*_eq_(C)] and O—H 0.82 Å (hydroxyl) [*U*_iso_(H) = 1.5*U*_eq_(O)].

### Crystal data

**Table:**

[Zn(C_9_H_8_N_3_O_6_S_3_)_2_(C_10_H_8_N_2_)_2_]	*Z* = 1
*M**_r_* = 1078.47	*F*(000) = 552
Triclinic, *P*1	*D*_x_ = 1.671 Mg m^−^^3^
Hall symbol: -P 1	Mo *K*α radiation, λ = 0.71073 Å
*a* = 8.6025 (7) Å	Cell parameters from 2836 reflections
*b* = 8.7606 (7) Å	θ = 2.5–28.1°
*c* = 15.3187 (12) Å	µ = 0.94 mm^−^^1^
α = 99.518 (1)°	*T* = 293 K
β = 105.802 (2)°	Block, colorless
γ = 98.805 (1)°	0.28 × 0.24 × 0.23 mm
*V* = 1071.41 (15) Å^3^	

### Data collection

**Table:**

Bruker SMART APEX CCD diffractometer	3745 independent reflections
Radiation source: fine-focus sealed tube	3103 reflections with *I* > 2σ(*I*)
graphite	*R*_int_ = 0.067
phi and ω scans	θ_max_ = 25.0°, θ_min_ = 2.4°
Absorption correction: multi-scan (*SADABS*; Sheldrick, 1996)	*h* = −8→10
*T*_min_ = 0.81, *T*_max_ = 0.84	*k* = −10→9
5465 measured reflections	*l* = −18→16

### Refinement

**Table:**

Refinement on *F*^2^	Primary atom site location: structure-invariant direct methods
Least-squares matrix: full	Secondary atom site location: difference Fourier map
*R*[*F*^2^ > 2σ(*F*^2^)] = 0.041	Hydrogen site location: inferred from neighbouring sites
*wR*(*F*^2^) = 0.116	H-atom parameters constrained
*S* = 1.05	*w* = 1/[σ^2^(*F*_o_^2^) + (0.0632*P*)^2^] where *P* = (*F*_o_^2^ + 2*F*_c_^2^)/3
3745 reflections	(Δ/σ)_max_ = 0.001
306 parameters	Δρ_max_ = 0.55 e Å^−^^3^
0 restraints	Δρ_min_ = −0.63 e Å^−^^3^

### Special details

**Table:**

Geometry. All esds (except the esd in the dihedral angle between two l.s. planes) are estimated using the full covariance matrix. The cell esds are taken into account individually in the estimation of esds in distances, angles and torsion angles; correlations between esds in cell parameters are only used when they are defined by crystal symmetry. An approximate (isotropic) treatment of cell esds is used for estimating esds involving l.s. planes.
Refinement. Refinement of *F*^2^ against ALL reflections. The weighted *R*-factor wR and goodness of fit *S* are based on *F*^2^, conventional *R*-factors *R* are based on *F*, with *F* set to zero for negative *F*^2^. The threshold expression of *F*^2^ > σ(*F*^2^) is used only for calculating *R*-factors(gt) etc. and is not relevant to the choice of reflections for refinement. *R*-factors based on *F*^2^ are statistically about twice as large as those based on *F*, and *R*- factors based on ALL data will be even larger.

### Fractional atomic coordinates and isotropic or equivalent isotropic displacement parameters (Å^2^)

**Table:**

	*x*	*y*	*z*	*U*_iso_*/*U*_eq_	
Zn1	0.5000	0.0000	0.5000	0.02725 (17)	
N1	0.1818 (3)	−0.1424 (3)	0.74960 (17)	0.0324 (6)	
N2	0.4260 (3)	−0.1972 (3)	0.71830 (15)	0.0265 (5)	
N3	0.4458 (3)	−0.0022 (3)	0.85212 (16)	0.0276 (6)	
N4	0.3822 (3)	0.1179 (3)	0.58899 (16)	0.0260 (5)	
N5	0.0151 (3)	0.5510 (3)	0.88027 (19)	0.0402 (7)	
O1	0.3126 (3)	−0.2111 (2)	0.48345 (15)	0.0365 (5)	
O2	0.3316 (3)	−0.4383 (2)	0.40496 (14)	0.0368 (5)	
H2	0.3378	−0.5271	0.4142	0.044*	
O3	0.6664 (2)	−0.0338 (2)	0.62169 (13)	0.0291 (5)	
O4	0.6623 (3)	−0.2886 (2)	0.57706 (14)	0.0352 (5)	
O5	0.1025 (3)	0.2799 (3)	1.01154 (16)	0.0456 (6)	
H5	0.0713	0.3389	1.0476	0.055*	
O6	0.3494 (3)	0.4151 (3)	1.09911 (19)	0.0634 (8)	
S1	0.13117 (9)	−0.35869 (9)	0.60274 (5)	0.0338 (2)	
S2	0.72472 (9)	−0.03437 (9)	0.82517 (5)	0.0308 (2)	
S3	0.17169 (10)	0.05612 (10)	0.89085 (6)	0.0401 (2)	
C1	0.2626 (3)	−0.2188 (3)	0.69989 (19)	0.0252 (6)	
C2	0.5103 (3)	−0.0856 (3)	0.79407 (19)	0.0247 (6)	
C3	0.2811 (4)	−0.0367 (3)	0.8256 (2)	0.0287 (7)	
C4	0.2717 (4)	−0.4370 (3)	0.5489 (2)	0.0267 (6)	
H4A	0.3749	−0.4300	0.5965	0.032*	
H4B	0.2259	−0.5481	0.5205	0.032*	
C5	0.3071 (4)	−0.3530 (3)	0.4759 (2)	0.0272 (6)	
C6	0.7620 (4)	−0.1775 (3)	0.73879 (19)	0.0282 (7)	
H6A	0.8804	−0.1675	0.7521	0.034*	
H6B	0.7170	−0.2824	0.7447	0.034*	
C7	0.6901 (3)	−0.1647 (3)	0.63890 (19)	0.0250 (6)	
C8	0.3335 (4)	0.2042 (4)	0.9770 (2)	0.0325 (7)	
H8A	0.4075	0.1532	1.0167	0.039*	
H8B	0.3968	0.2662	0.9465	0.039*	
C9	0.2604 (4)	0.3118 (4)	1.0352 (2)	0.0346 (7)	
C10	0.2189 (4)	0.1151 (4)	0.5617 (2)	0.0334 (7)	
H10	0.1555	0.0562	0.5031	0.040*	
C11	0.1411 (4)	0.1955 (4)	0.6167 (2)	0.0329 (7)	
H11	0.0278	0.1896	0.5950	0.039*	
C12	0.2330 (4)	0.2856 (3)	0.7048 (2)	0.0269 (6)	
C13	0.4010 (4)	0.2880 (3)	0.7317 (2)	0.0309 (7)	
H13	0.4681	0.3470	0.7896	0.037*	
C14	0.4687 (4)	0.2032 (3)	0.6729 (2)	0.0297 (7)	
H14	0.5815	0.2061	0.6932	0.036*	
C15	0.1554 (4)	0.3774 (3)	0.7653 (2)	0.0277 (6)	
C16	−0.0110 (4)	0.3372 (4)	0.7560 (2)	0.0388 (8)	
H16	−0.0784	0.2503	0.7110	0.047*	
C17	−0.0762 (4)	0.4272 (4)	0.8142 (2)	0.0447 (9)	
H17	−0.1885	0.3999	0.8068	0.054*	
C18	0.1757 (4)	0.5904 (4)	0.8904 (2)	0.0383 (8)	
H18	0.2401	0.6770	0.9367	0.046*	
C19	0.2497 (4)	0.5074 (4)	0.8345 (2)	0.0352 (7)	
H19	0.3622	0.5382	0.8431	0.042*	

### Atomic displacement parameters (Å^2^)

**Table:**

	*U*^11^	*U*^22^	*U*^33^	*U*^12^	*U*^13^	*U*^23^
Zn1	0.0323 (3)	0.0258 (3)	0.0252 (3)	0.0085 (2)	0.0130 (2)	0.0003 (2)
N1	0.0275 (14)	0.0362 (14)	0.0309 (14)	0.0065 (11)	0.0110 (11)	−0.0026 (11)
N2	0.0244 (13)	0.0290 (13)	0.0244 (13)	0.0052 (11)	0.0077 (10)	0.0012 (11)
N3	0.0256 (13)	0.0322 (13)	0.0241 (13)	0.0078 (11)	0.0089 (10)	−0.0001 (11)
N4	0.0286 (13)	0.0230 (12)	0.0266 (13)	0.0066 (10)	0.0109 (11)	0.0013 (10)
N5	0.0369 (16)	0.0439 (16)	0.0404 (16)	0.0147 (13)	0.0159 (13)	−0.0018 (13)
O1	0.0389 (13)	0.0255 (12)	0.0460 (13)	0.0062 (9)	0.0162 (10)	0.0056 (10)
O2	0.0511 (14)	0.0299 (11)	0.0338 (12)	0.0098 (11)	0.0192 (10)	0.0071 (10)
O3	0.0352 (12)	0.0253 (10)	0.0276 (11)	0.0089 (9)	0.0110 (9)	0.0028 (9)
O4	0.0466 (14)	0.0284 (11)	0.0301 (12)	0.0123 (10)	0.0124 (10)	0.0000 (9)
O5	0.0359 (14)	0.0519 (15)	0.0437 (14)	0.0148 (11)	0.0142 (11)	−0.0129 (11)
O6	0.0462 (16)	0.0648 (17)	0.0614 (18)	0.0071 (14)	0.0157 (13)	−0.0295 (14)
S1	0.0239 (4)	0.0386 (5)	0.0307 (4)	−0.0020 (3)	0.0090 (3)	−0.0085 (3)
S2	0.0220 (4)	0.0406 (5)	0.0236 (4)	0.0047 (3)	0.0050 (3)	−0.0051 (3)
S3	0.0272 (4)	0.0481 (5)	0.0387 (5)	0.0078 (4)	0.0134 (4)	−0.0135 (4)
C1	0.0253 (15)	0.0254 (15)	0.0257 (15)	0.0063 (12)	0.0099 (12)	0.0032 (12)
C2	0.0250 (15)	0.0265 (15)	0.0222 (14)	0.0062 (12)	0.0070 (12)	0.0034 (12)
C3	0.0281 (16)	0.0310 (16)	0.0275 (16)	0.0085 (13)	0.0107 (13)	0.0022 (13)
C4	0.0273 (15)	0.0241 (14)	0.0272 (15)	0.0048 (12)	0.0096 (12)	−0.0005 (12)
C5	0.0266 (16)	0.0238 (16)	0.0294 (16)	0.0050 (12)	0.0085 (12)	0.0013 (12)
C6	0.0248 (15)	0.0309 (16)	0.0289 (16)	0.0088 (13)	0.0090 (12)	0.0023 (13)
C7	0.0232 (15)	0.0267 (15)	0.0259 (15)	0.0057 (12)	0.0113 (12)	0.0013 (12)
C8	0.0281 (16)	0.0381 (17)	0.0300 (16)	0.0106 (14)	0.0094 (13)	−0.0002 (14)
C9	0.0316 (18)	0.0373 (18)	0.0320 (17)	0.0084 (14)	0.0100 (14)	−0.0018 (14)
C10	0.0335 (17)	0.0319 (16)	0.0318 (17)	0.0054 (14)	0.0117 (14)	−0.0030 (13)
C11	0.0290 (16)	0.0347 (17)	0.0329 (17)	0.0068 (13)	0.0112 (13)	−0.0013 (14)
C12	0.0293 (16)	0.0259 (15)	0.0274 (15)	0.0074 (12)	0.0118 (12)	0.0044 (12)
C13	0.0308 (17)	0.0325 (16)	0.0279 (16)	0.0080 (14)	0.0098 (13)	−0.0003 (13)
C14	0.0293 (16)	0.0312 (16)	0.0290 (16)	0.0094 (13)	0.0097 (13)	0.0033 (13)
C15	0.0287 (16)	0.0298 (15)	0.0258 (15)	0.0099 (13)	0.0099 (12)	0.0034 (12)
C16	0.0355 (18)	0.0409 (19)	0.0383 (19)	0.0095 (15)	0.0137 (15)	−0.0015 (15)
C17	0.0361 (19)	0.052 (2)	0.046 (2)	0.0134 (17)	0.0169 (16)	−0.0018 (17)
C18	0.0390 (19)	0.0377 (18)	0.0368 (18)	0.0103 (15)	0.0142 (15)	−0.0026 (15)
C19	0.0319 (17)	0.0356 (17)	0.0378 (18)	0.0089 (14)	0.0148 (14)	−0.0018 (14)

### Geometric parameters (Å, °)

**Table:**

Zn1—O3^i^	2.1145 (19)	S3—C3	1.739 (3)
Zn1—O3	2.1145 (19)	S3—C8	1.800 (3)
Zn1—N4^i^	2.135 (2)	C4—C5	1.507 (4)
Zn1—N4	2.135 (2)	C4—H4A	0.9700
Zn1—O1	2.189 (2)	C4—H4B	0.9700
Zn1—O1^i^	2.189 (2)	C6—C7	1.516 (4)
N1—C1	1.331 (4)	C6—H6A	0.9700
N1—C3	1.342 (4)	C6—H6B	0.9700
N2—C1	1.333 (4)	C8—C9	1.513 (4)
N2—C2	1.334 (3)	C8—H8A	0.9700
N3—C3	1.334 (4)	C8—H8B	0.9700
N3—C2	1.346 (4)	C10—C11	1.382 (4)
N4—C14	1.325 (4)	C10—H10	0.9300
N4—C10	1.347 (4)	C11—C12	1.394 (4)
N5—C17	1.327 (4)	C11—H11	0.9300
N5—C18	1.331 (4)	C12—C13	1.386 (4)
O1—C5	1.222 (3)	C12—C15	1.486 (4)
O2—C5	1.299 (3)	C13—C14	1.379 (4)
O2—H2	0.8200	C13—H13	0.9300
O3—C7	1.252 (3)	C14—H14	0.9300
O4—C7	1.261 (3)	C15—C16	1.383 (4)
O5—C9	1.279 (4)	C15—C19	1.388 (4)
O5—H5	0.8200	C16—C17	1.380 (4)
O6—C9	1.204 (4)	C16—H16	0.9300
S1—C1	1.747 (3)	C17—H17	0.9300
S1—C4	1.795 (3)	C18—C19	1.379 (4)
S2—C2	1.740 (3)	C18—H18	0.9300
S2—C6	1.797 (3)	C19—H19	0.9300
			
O3^i^—Zn1—O3	180.00 (7)	C7—C6—H6A	108.4
O3^i^—Zn1—N4^i^	86.91 (8)	S2—C6—H6A	108.4
O3—Zn1—N4^i^	93.09 (8)	C7—C6—H6B	108.4
O3^i^—Zn1—N4	93.09 (8)	S2—C6—H6B	108.4
O3—Zn1—N4	86.91 (8)	H6A—C6—H6B	107.4
N4^i^—Zn1—N4	180.00 (9)	O3—C7—O4	123.7 (3)
O3^i^—Zn1—O1	84.73 (8)	O3—C7—C6	119.5 (2)
O3—Zn1—O1	95.27 (8)	O4—C7—C6	116.8 (2)
N4^i^—Zn1—O1	94.10 (8)	C9—C8—S3	110.2 (2)
N4—Zn1—O1	85.90 (8)	C9—C8—H8A	109.6
O3^i^—Zn1—O1^i^	95.27 (8)	S3—C8—H8A	109.6
O3—Zn1—O1^i^	84.73 (8)	C9—C8—H8B	109.6
N4^i^—Zn1—O1^i^	85.90 (8)	S3—C8—H8B	109.6
N4—Zn1—O1^i^	94.10 (8)	H8A—C8—H8B	108.1
O1—Zn1—O1^i^	180.00 (7)	O6—C9—O5	125.3 (3)
C1—N1—C3	113.8 (3)	O6—C9—C8	120.2 (3)
C1—N2—C2	113.8 (2)	O5—C9—C8	114.4 (3)
C3—N3—C2	113.4 (2)	N4—C10—C11	123.2 (3)
C14—N4—C10	116.8 (3)	N4—C10—H10	118.4
C14—N4—Zn1	121.0 (2)	C11—C10—H10	118.4
C10—N4—Zn1	122.14 (19)	C10—C11—C12	119.7 (3)
C17—N5—C18	118.6 (3)	C10—C11—H11	120.1
C5—O1—Zn1	136.6 (2)	C12—C11—H11	120.1
C5—O2—H2	109.5	C13—C12—C11	116.5 (3)
C7—O3—Zn1	125.67 (18)	C13—C12—C15	121.9 (3)
C9—O5—H5	109.5	C11—C12—C15	121.7 (3)
C1—S1—C4	103.09 (14)	C14—C13—C12	120.1 (3)
C2—S2—C6	100.14 (13)	C14—C13—H13	119.9
C3—S3—C8	101.90 (14)	C12—C13—H13	119.9
N1—C1—N2	126.4 (3)	N4—C14—C13	123.7 (3)
N1—C1—S1	113.1 (2)	N4—C14—H14	118.1
N2—C1—S1	120.6 (2)	C13—C14—H14	118.1
N2—C2—N3	126.3 (3)	C16—C15—C19	117.5 (3)
N2—C2—S2	120.1 (2)	C16—C15—C12	122.0 (3)
N3—C2—S2	113.6 (2)	C19—C15—C12	120.5 (3)
N3—C3—N1	126.3 (3)	C17—C16—C15	119.4 (3)
N3—C3—S3	121.1 (2)	C17—C16—H16	120.3
N1—C3—S3	112.6 (2)	C15—C16—H16	120.3
C5—C4—S1	113.8 (2)	N5—C17—C16	122.6 (3)
C5—C4—H4A	108.8	N5—C17—H17	118.7
S1—C4—H4A	108.8	C16—C17—H17	118.7
C5—C4—H4B	108.8	N5—C18—C19	122.3 (3)
S1—C4—H4B	108.8	N5—C18—H18	118.9
H4A—C4—H4B	107.7	C19—C18—H18	118.9
O1—C5—O2	121.7 (3)	C18—C19—C15	119.6 (3)
O1—C5—C4	121.3 (3)	C18—C19—H19	120.2
O2—C5—C4	116.9 (2)	C15—C19—H19	120.2
C7—C6—S2	115.7 (2)		

Symmetry codes: (i) −*x*+1, −*y*, −*z*+1.

### Hydrogen-bond geometry (Å, °)

**Table:**

*D*—H···*A*	*D*—H	H···*A*	*D*···*A*	*D*—H···*A*
O2—H2···O4^ii^	0.82	1.64	2.460 (3)	175
O5—H5···N5^iii^	0.82	1.74	2.554 (3)	174
C13—H13···O6^iv^	0.93	2.47	3.335 (4)	156
C19—H19···O6^iv^	0.93	2.34	3.245 (4)	164
C6—H6A···N1^v^	0.97	2.58	3.533 (4)	168

Symmetry codes: (ii) −*x*+1, −*y*−1, −*z*+1; (iii) −*x*, −*y*+1, −*z*+2; (iv) −*x*+1, −*y*+1, −*z*+2; (v) *x*+1, *y*, *z*.
